# Polygonum Hydropiper*Arranged by Dr. Edward Bayard.

**Published:** 1885-09

**Authors:** 


### POLYGONUM HYDROPIPER*

(SMART WEED).

MIND AND DISPOSITION.—*Great depression, followed by excessive irritability.* Gloomy views of life and dislike of change and excessive dread of death.

HEAD.—Dizziness. Pressure in back of head, on lying down. Pain upon sudden rising in back of head, with pain over eyes. These symptoms are much increased in damp weather, disappearing in moderately warm temperature. Acute pain in left side of face, extending to the temple, sometimes darting through whole left side of head. Under pressure of great weariness or excitement, a dull, depressed pain through the whole head, causing a sensation of torpor and a strong desire to sleep, with inability to. A sensation of sudden rising of scalp, with extreme irritation and increased dry exfoliation.

EYES.—Burning sensation in eyeballs and dry sensation in lids. Convulsive twitching of lids, when closed and when lying down, dizziness and wavering sight. Inflammation of edges of lids.

EARS.—Dullness of hearing. Ringing sounds in ears. Sudden sounds on tympanum, producing momentary cessation of hearing. Acute pain in ear, when bending head down; relieved by bending head backward. Moist atmosphere aggravating all these symptoms. Secretion of ears increased.

NOSE.—Inflammation of mucous membrane of nose. Tickling sensation in nostrils. Reddened or inflamed appearance of nostrils.

CORYZA.—Inflammation of mucous membrane of eyes. Colorless liquid from eyes and nostrils, with a swollen sensation in both. Feeling of congestion through eyes and nose. Coldness in exterior of nose.

FACE.—Excruciating pain and heat in left side of face, much increased by cold or damp. Coldness in right side of face, when pain is most severe on left. After severe pain, sometimes drawing in left side of face, from chin to temple; this is accompanied by paleness, with a drawn, tired look.

TEETH.—A soreness on pressure, with a sensation of enlargement. Increase temperature in mouth, leaving this sensation.

GUMS.—Tenderness of gums. Cold or cooling temperature in mouth, producing an acute toothache.

---

*Arranged by Dr. Edward Bayard.

MOUTH.—Bitter taste in mouth. Heat in roof of mouth, with excitation of salivary glands. An increased flow of heated saliva, affording no relief to the parched condition. Throat dry and hot with sensation of excoriation. Glands feel swollen, cold or moist air increasing it.

APPETITE AND TASTE.—Bitter taste. Loss of appetite. Food is tasteless or nearly so. Repulsion to swallowing. A contracted feeling in throat after swallowing followed by thirst. No relief after drinking.

STOMACH.—Feeling of weight in. *Pressure of clothes causing distress.* Slight acidity. Pain upon pressure, followed by throbbing and distress. General uneasiness in stomach and abdomen. Abdomen, pain in.

STOOL.—Dark discharges followed by burning sensation in rectum. All these symptoms increased on lying down. Occasionally burning heat in both stomach and bowels. Cold temperature increasing both evacuations and violence of pain.

URINE.—When the kidneys are affected by a cold causing a great disturbance of the secretions, such as inflammation, urine high colored, dark red, and in diseases of the kidneys themselves, its action is specific. Causes an arrest of albumen, a deposition of mucus in the bladder, and of the phosphates. With disease of the kidneys, pain in the back and at the lower extremity, at times acute, and again a drawing pain, lateral in its action—AS IF THE HIPS WERE BEING DRAWN TOGETHER.

LOINS.—Tearing and drawing sensation in, and on exposure to cold, followed by lameness and soreness of loins.

MALE GENITAL ORGANS.—Irritation of genital organs. Loss of power and of semen, sometimes followed by inflammation of glans penis. An extremely convulsive action in their functional uses.

FEMALE GENITAL ORGANS.—Congestive weakness and loss of power. Intense dislike for coition, followed by a perturbation and irritation if approached. Inaction of the flow of the secretions.

MENSES.—Congestion of ovaries. Tearing sensation in groin, especially right. Tardy menstruation from inaction and too copious, amounting almost to hemorrhage. Pressure and soreness in head. Grinding pain through abdomen. In case of too tardy menstruation, the secretions become fetid, discharging offensive matter. Acid leucorrhœa, excoriating the passages. It also produces a violent virulent action of the ovaries, followed by nausea, vertigo, and complete prostration. In connection with these conditions the respiratory organs are much affected. Misconception.

CHEST.—The movement of the abdomen in breathing becomes painful. Lungs enfeebled and breathing labored. Increased action of heart, with loss of rhythm. *Shooting pains through mammæ with great soreness of same.* Hardness, distention, and great tenderness of mammæ.

LARYNX.—Stifling sensation in, and an irritability of the whole system; with this disturbance a weakness of the functions of the genital organs. Crowding and pressure about larynx, with irritation of the bronchi. Hacking cough, more strongly manifest by a change of temperature. Sometimes a roughness as of adhesion of mucus to larynx, producing a spasmodic hacking and hoarseness.

EXTREMITIES.—Sensation of weakness in both upper and lower. Distention of blood-vessels in both hands and feet. Swelling of both legs and feet. Pain in arms, and inability or sense of weakness in lifting the slightest weight.

